# Comparative morphology and transcriptome analysis reveals distinct functions of the primary and secondary laticifer cells in the rubber tree

**DOI:** 10.1038/s41598-017-03083-3

**Published:** 2017-06-09

**Authors:** Deguan Tan, Xiaowen Hu, Lili Fu, Anuwat Kumpeangkeaw, Zehong Ding, Xuepiao Sun, Jiaming Zhang

**Affiliations:** 1 Institute of Tropical Bioscience and Biotechnology, MOA Key Laboratory of Tropical Crops Biology and Genetic Resources, Hainan Bioenergy Center, CATAS, Xueyuan Road 4, Haikou, Hainan Province 571101 China; 2 Zhanjiang Experimental Station, CATAS, West Libration Road 20, Zhanjiang, Guangdong Province 524013 China; 3 Song Khla Rubber Research Centre, Department of Agriculture, Ministry of Agriculture and Cooperatives, Had Yai, Song Khla 90110 Thailand

## Abstract

Laticifers are highly specialized cells that synthesize and store natural rubber. Rubber trees (*Hevea brasiliensis* Muell. Arg.) contain both primary and secondary laticifers. Morphological and functional differences between the two types of laticifers are largely unknown, but such information is important for breeding and cultivation practices. Morphological comparison using paraffin sections revealed only distribution differences: the primary laticifers were distributed randomly, while the secondary laticifers were distributed in concentric rings. Using isolated laticifer networks, the primary laticifers were shown to develop via intrusive “budding” and formed necklace-like morphology, while the secondary laticifers developed straight and smooth cell walls. Comparative transcriptome analysis indicated that genes involved in cell wall modification, such as pectin esterase, lignin metabolic enzymes, and expansins, were highly up-regulated in the primary laticifers and correspond to its necklace-like morphology. Genes involved in defense against biotic stresses and rubber biosynthesis were highly up-regulated in the primary laticifers, whereas genes involved in abiotic stresses and dormancy were up-regulated in the secondary laticifers, suggesting that the primary laticifers are more adequately prepared to defend against biotic stresses, while the secondary laticifers are more adequately prepared to defend against abiotic stresses. Therefore, the two types of laticifers are morphologically and functionally distinct.

## Introduction

Natural rubber consists primarily of isoprene (cis-1,4-polyisoprene) polymers, and is used in a diverse products, such as tires, condoms, shoes, dampening and insulating elements, and approximately 40,000 other products^1^. Due primarily to its molecular structure and high molecular weight (>1 million Da)^2–4^, natural rubber has a large stretch ratio, high resilience, abrasion resistance, efficient heat dispersion, impact resistance, and extreme waterproof characteristics. This combination of high-performance properties cannot easily be mimicked by artificially produced polymers^1, 5^, and natural rubber still accounts for more than 30% of the 15 billion kilograms of rubber produced annually^6^.

Natural rubber is synthesized in highly specialized plant cells, known as laticifers or latex vessels. Approximately 12,500 plant species among 22 families produce rubber in laticifers^7^; however, the rubber tree (*Hevea brasiliensis* Muell. Arg.) is the only source of commercial natural rubber in the global market^8, 9^. The economic importance of natural rubber has prompted investigations into its biosynthesis. Both the primary and the secondary laticifers in the rubber tree produce rubber, but only the secondary laticifers in the bark of mature trees are economically exploited. Primary laticifers are not tapped in production practice, because the primary laticifers are mainly present in the bark of young shoots or stems but disappear in the bark of the tree trunk during maturation (>6 years old)^10^. The primary laticifers are differentiated from the meristematic zones, while the secondary laticifers are differentiated from vascular cambium and are present in rings parallel to the vascular cambium in the secondary phloem of the trunk.

The number of secondary laticifer rings varies in different rubber tree clones, which are propagated by bud grafting and are used for commercial cultivation. Clones with more secondary laticifer rings typically have a higher rubber yield^8, 11, 12^. Therefore, the secondary laticifers have become the focus of rubber tree researchers. By comparison, less research has focused on the primary laticifer cells. The number and latex-producing potential of the primary laticifer cells have been used for early predictions of rubber yield of hybrid clones to shorten the breeding cycle^13, 14^; however, these predictions have been reported to be inaccurate^12, 15^. The relationship and/or correlation between the primary and the secondary laticifers are important for breeding and cultivation practices, but these relationships need to be thoroughly investigated. In this study, we compared the morphological differences of the primary and the secondary laticifers by investigating isolated laticifer networks using a new technology instead of conventional paraffin sections, and revealed significant morphological differences. Additionally, comparative transcriptome analysis revealed functional differences between the two types of laticifers.

## Results

### Morphological comparison of the primary and the secondary laticifers in rubber tree

**Figure 1:**
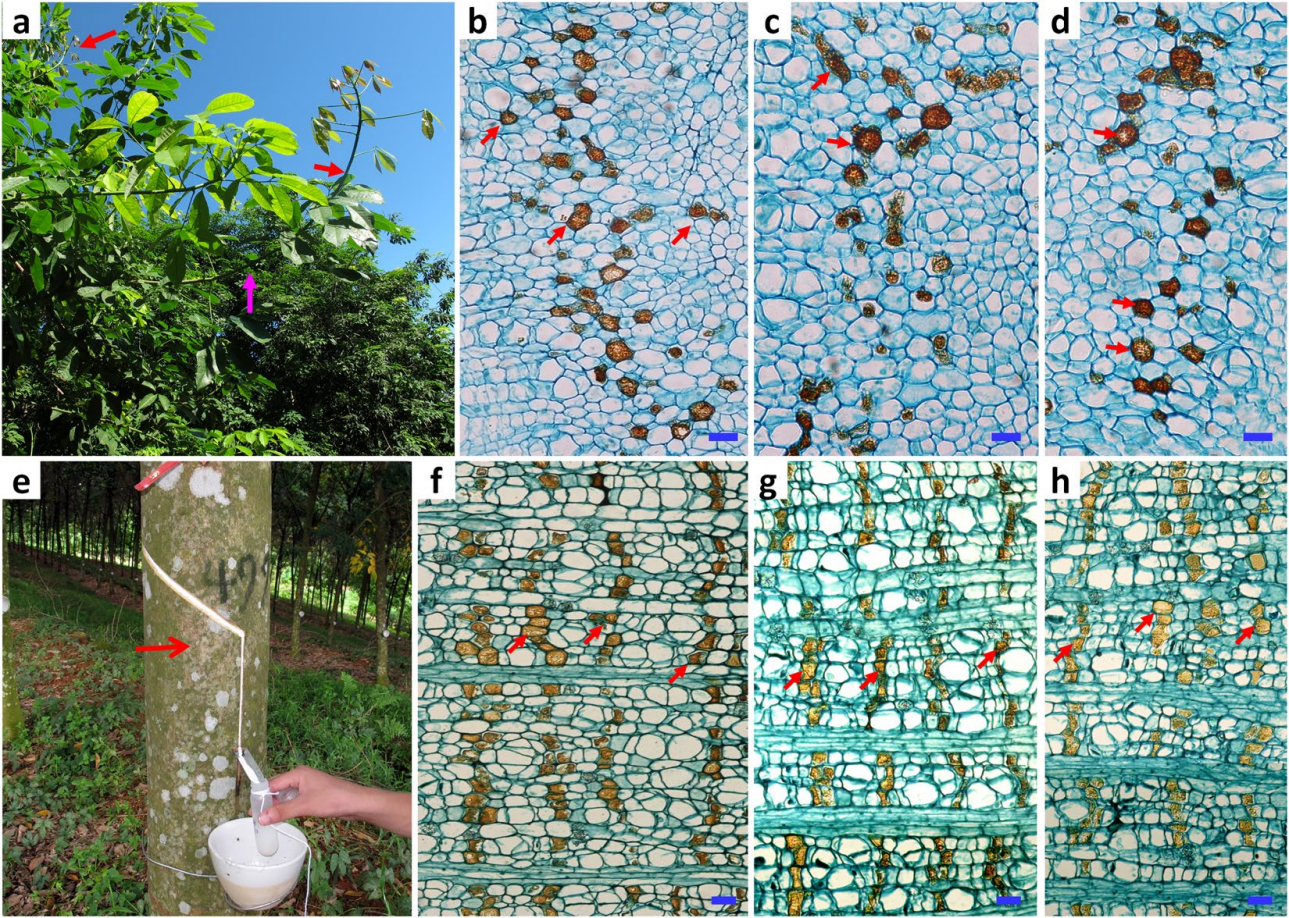
A comparison of the primary and the secondary laticifer cells in cross-sections. (**a**) Canopy of rubber tree clone RY7-33-97 (7-years old). Red arrows indicate the young shoots where the primary latex for RNA isolation and the bark samples for histological observation were collected, and the pink arrow indicates the region containing both the primary and the secondary laticifer cells. (**b**–**d**) Representative cross-sections of the bark of young shoots selected from three biological replicates. Red arrows indicate the primary laticifer cells. The scale bar represents 20 µm; (**e**) A rubber tree trunk showing the latex in the first tapping. The red arrow indicates where the bark sample was collected for anatomical analysis. (**f**–**h**) Representative cross-sections of the bark from tree trunks showing the secondary laticifer cells; The scale bar represents 20 µm.

The newly emerged young shoots of rubber trees contained only the primary laticifer cells (Fig. 1a–d), while the tree trunk contained only the secondary laticifers (Fig. 1e–h). Following staining of cross-sections with iodine bromide solution, no obvious morphological differences between single cells of the primary and the secondary laticifers were observed, except for the distribution pattern. The primary laticifer cells were distributed more or less at random in the phloem (Fig. 1b–d). In contrast, the secondary laticifer cells were distributed in rows and formed multiple rings around the trunk (Fig. 1f–h).

**Figure 2:**
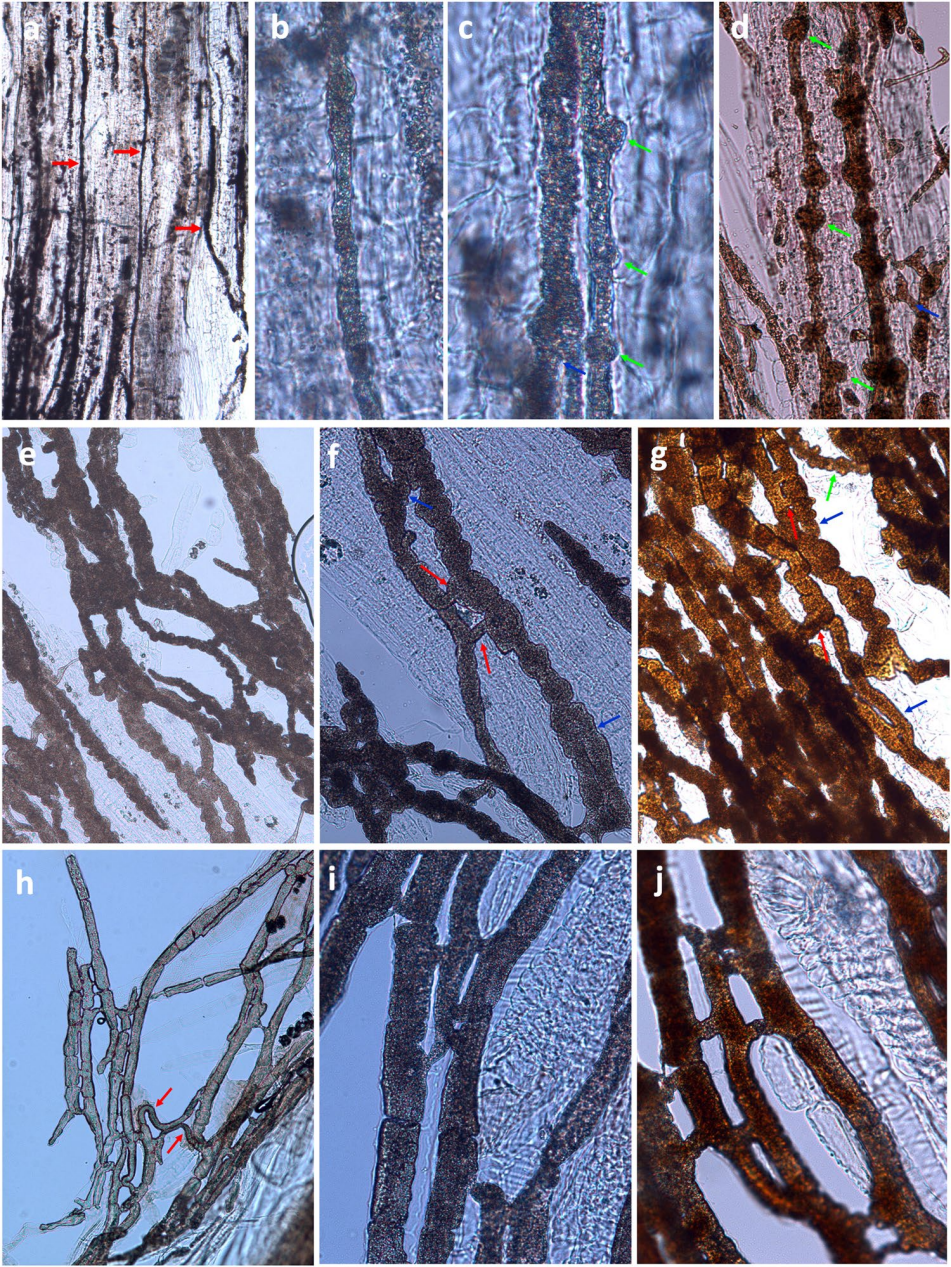
Laticifer cells partially isolated from the rubber tree bark. (**a**–**g**) Primary laticifers unstained (**a**–**c** and **e**–**f**) or stained with iodine and bromine solution (**d**,**g**); (**h**–**j**) secondary laticifer cells unstained (**h** and **i**) or stained with iodine and bromine solution (**j**). Yellow arrows indicate primary laticifers; green arrows indicate intrusive growth by “budding;” blue arrows indicate laticifer branches; red arrows indicate linkage tubes between two laticifers.

The primary and the secondary laticifer networks were partially isolated from bark, along with a few parenchyma cells to be used as reference cells (Fig. 2). The laticifer cells were easily identified by their dark color due to the rubber content in the absence of staining. The primary laticifer cells were differentiated from meristematic cells, and the initial laticifer tubes were thin, straight, un-branched, and often non-articulated (Fig. 2a,b). During development, primary laticifer tubes initiated intrusive growth and expanded towards the neighboring parenchyma cells at discrete locations (Fig. 2c), and eventually, the tubes formed necklace-like structures (Fig. 2d,e). Laticifer branches were also formed at this time (Fig. 2c,d). The tubes were then inflated at additional locations and formed dense necklace-like structures (Fig. 2e). The neighboring “beads” were merged gradually to form laticifer tubes of larger diameter, and ultimately, neighboring tubes were connected by bridges to form a laticifer network (Fig. 2e,f,g).

The secondary laticifer cells originated from cambium cells. These laticifers were articulated and had thick, straight, and smooth cell walls (Fig. 2h,i,j). The neighboring tubes were anastomosed by short bridges, and some distant and parallel tubes were joined by specialized laticifer cells that grew transversely in the bark (Fig. 2h). Each cell was joined to two or more cells of the neighboring tubes at different locations (Fig. 2h,i,j).

### Sequencing, assembling, and enrichment analysis of differentially expressed genes (DEGs)

**Figure 3:**
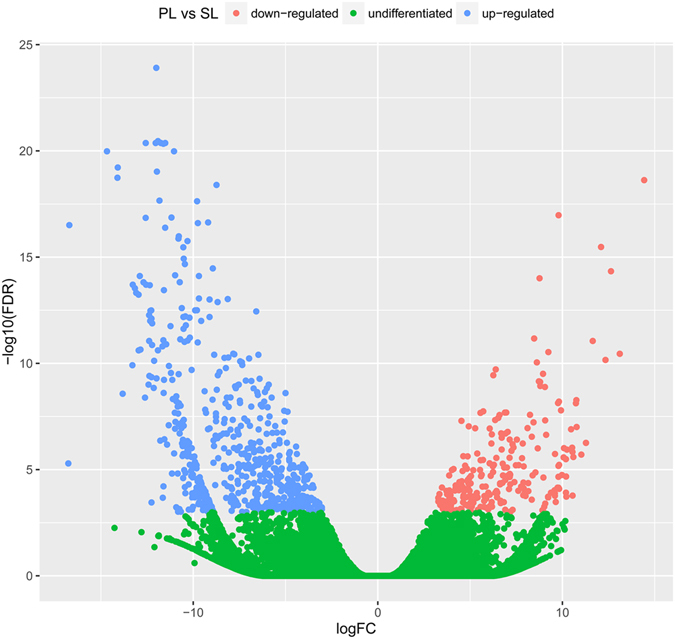
Differential expression analysis of unigenes in the primary laticifers (PL) versus the secondary laticifers (SL). The LogFC (log2 Fold Change) is used as the x-axis and –log10FDR (False Discovery Rate) is used as the y-axis. The total number of unigenes is 70,933. Of those 70,089 were not DEGs (cut-off FDR ≤0.001, highlighted in green), 597 unigenes were up-regulated in PL (highlighted in blue), and 247 unigenes were down-regulated (highlighted in red).

To understand the distinct morphology of the primary and the secondary laticifer vessels, the transcriptomes of these cell types of virgin RY7-33-97 rubber trees were sequenced using the Illumina paired-end sequencing method with three biological replicates. Totally six RNA-seq libraries were generated and more than 312 million raw reads were obtained. After trimming adaptors and removing reads with low quality, 262 million (84.0%) clean reads were remained and were used to construct de novo transcription profiles with the Trinity software^16, 17^. In total, 131,078 transcripts (N50 = 1,406) and 103,704 unigenes were identified (Supplementary Table S1). Sample clustering of gene expression indicated that replicates of the same tissue were grouped together (Supplementary Fig. S1). PCA plot analysis indicated that the replicates of PL and SL samples were well separated (Supplementary Fig. S2). These results support the uniformity and reliability of the transcriptome data across replicates. The gene expression data was normalized with the trimmed mean of M values (TMM) method and the abundance was calculated as transcripts per million transcripts (TPM). Filtering of the expressed genes with TPM ≤1, we identified 70,933 unigenes. A total of 48,780 (68.77%) unigenes were hit and functionally annotated by BLAST against publicly available databases using a cutoff E-value < 1.00E-5 (Supplementary Table S2), in which the databases SwissProt, nt, GO, and TrEMBL accounted for 39.68%, 50.29%, 41.02%, and 63.29% of the annotation, respectively (Supplementary Table S3). In total, 844 DEGs were identified using edgeR, an R program package, with an adjusted *p*-value cut-off for false discovery rate (FDR) ≤0.001 and |log2Ratio| ≥2^18^. Of these DEGs, 597 were up-regulated and 247 were down-regulated in the primary laticifers compared to the levels in the secondary laticifers (Fig. 3), indicating that the gene expression profiles were significantly changed during the transition of these two types of laticifer cells.

To gain insight into the pathways that were altered during the transition between the primary and the secondary laticifers, the 844 DEGs were annotated and imported to MapMan for visualization. This analysis revealed that most genes involved in cell wall synthesis, degradation, and modification were up-regulated as were genes involved in lignin biosynthesis, amino acid metabolism, redox, and secondary metabolism of isoprenoids, while genes related to trehalose biosynthesis, lipid degradation, and gluconeogenesis were down-regulated in the primary laticifers compared to the secondary laticifers (Supplementary Fig. S3). Gene ontology (GO) analysis also showed similar results (Supplementary Table S4). In total, 142 enriched GO categories were identified, and 94 of these were enriched in the primary laticifers, while 50 were enriched in the secondary laticifers (Supplementary Table S4). Two of these (GO:0050267, rubber cis-polyprenylcistransferase activity; GO:0080167, response to karrikin) were commonly enriched in both the primary and the secondary laticifers (Supplementary Table S4).

### Genes involved in cell development and cell wall modification are up-regulated in the primary laticifers

**Figure 4:**
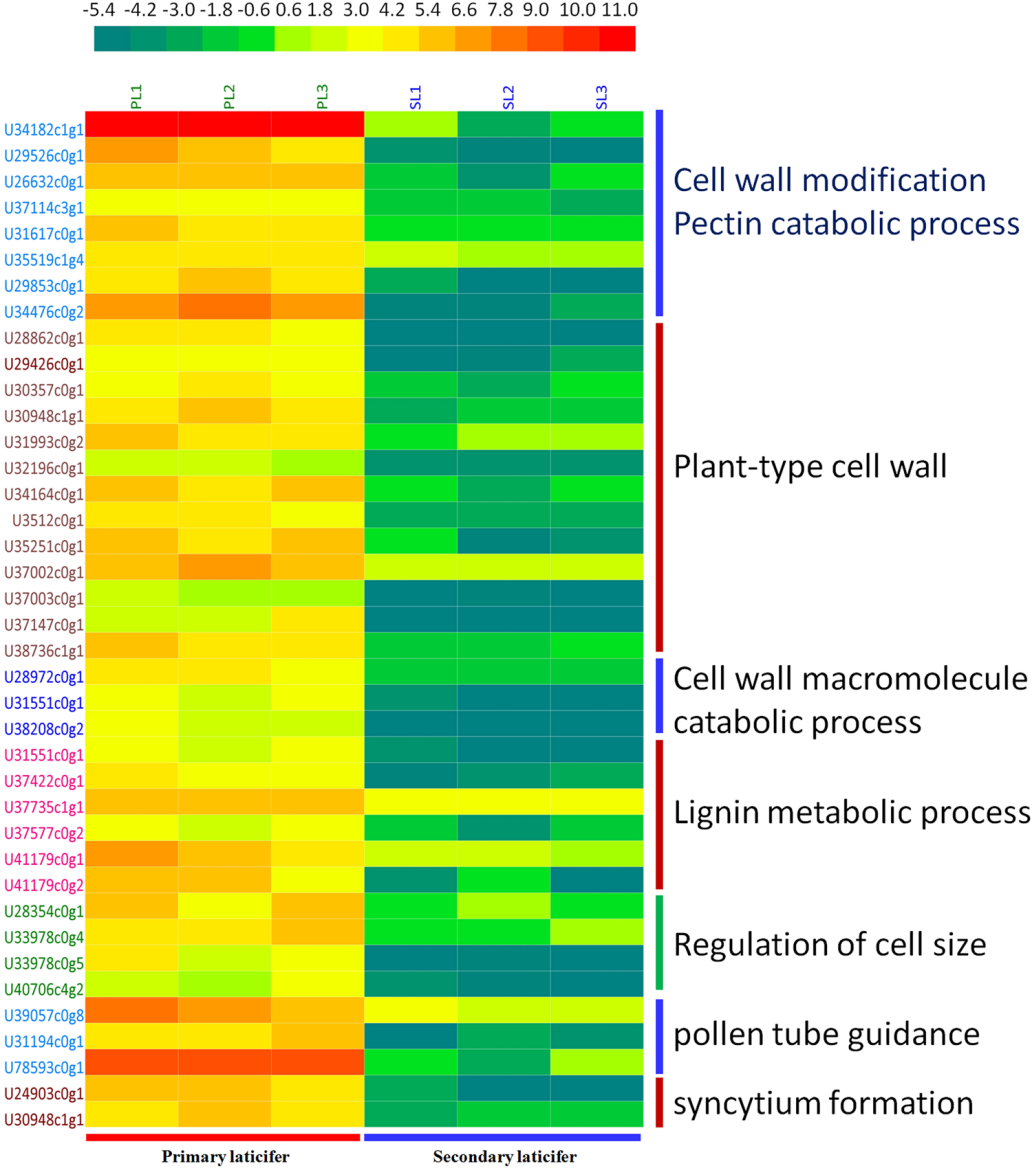
Heatmap of DEGs involved in cell growth and cell wall modification. The expression levels of DEGs in TPM were compiled with Excel 2007, normalized using a log2 base method, and exported to the HemI toolkit^124^. The Heatmap was generated using the default parameters. The expression levels are presented as different colors, and the values are defined in the scale bar. PL, the primary laticifers; SL, the secondary laticifers.

DEGs involved in cell development and cell wall modification were up-regulated in the primary laticifers (Supplementary Table S4). These genes were clustered into the sub-categories cell wall modification, pectin catabolic process, plant-type cell wall, cell wall macromolecule catabolic process, lignin metabolic process, regulation of cell size, pollen tube guidance, and syncytium formation (Fig. 4). The most significant DEGs belonged to pectin catabolic processes and included eight unigenes that encode pectinesterase (Fig. 4). Of these eight, U34182c1g1 was the most differentially expressed. The TPM values of U34182c1g1 in the primary and the secondary laticifers were 4,294 and 1, respectively. Pectinesterase is a ubiquitous cell-wall-associated enzyme that facilitates pectin de-esterification, cell wall modification, and subsequent breakdown^19, 20^. The over-expression of pectinesterases in the primary laticifers may play a major role in intrusive growth, including the formation of necklace-like structures and subsequent cell expansion of the primary laticifers (Fig. 2).

A total of thirteen DEGs that were up-regulated in the primary laticifers were clustered into plant-type cell wall (Fig. 4). These DEGs encoded berberine bridge enzymes, methyltransferases, class III peroxidases, alpha-expansin, RALF-like protein, subtilisin-like protease, pectin acetylesterase, alpha-glucosidase, vacuolar invertase, and heparanase-like protein. Six DEGs were clustered in the lignin metabolic process and included laccase, transaldolase, chitinase-like protein, and three xylem serine proteinase genes. Four DEGs were clustered in the regulation of cell size, and these genes encoded O-glycosyl hydrolases family 17, two metallothionein 3-like proteins, and WALLS ARE THIN 1 (WAT1)-like protein. Other DEGs that were up-regulated in the primary laticifers included three DEGs in the cell wall macromolecule catabolic process and three DEGs in the pollen tube guidance and regulation of pollen growth. An annexin-like protein (U39057c0g8) with homologs that have been implicated in regulation of pollen tube and fiber growth^21, 22^ in Arabidopsis and cotton, respectively, was highly up-regulated in the primary laticifers. Annexins interact with cell membrane components that are relevant to the structural organization of the cell, and these proteins are involved in cell shape, intracellular signaling, and growth control and act as atypical calcium channels^23, 24^. Therefore, the growth of the primary laticifer cells may share some similarities with pollen tubes and fibers.

Interestingly, two DEGs involved in syncytium formation were up-regulated in the primary laticifer (Fig. 4). One (U24903c0g1) encoded an expansin B3-like protein, and the other encoded an alpha-expansin 4-like protein. Expansins are involved in extensive cell wall modification during interactions between nematodes and host^25–28^.

Taken together, numerous DEGs that are related to cell wall modification and/or cell development were up-regulated in the primary laticifers, resulting in the active intrusive growth of the primary laticifer cells and the formation of their necklace-like morphology.

### Genes involved in defense against biotic stresses are up-regulated in the primary laticifer

**Figure 5:**
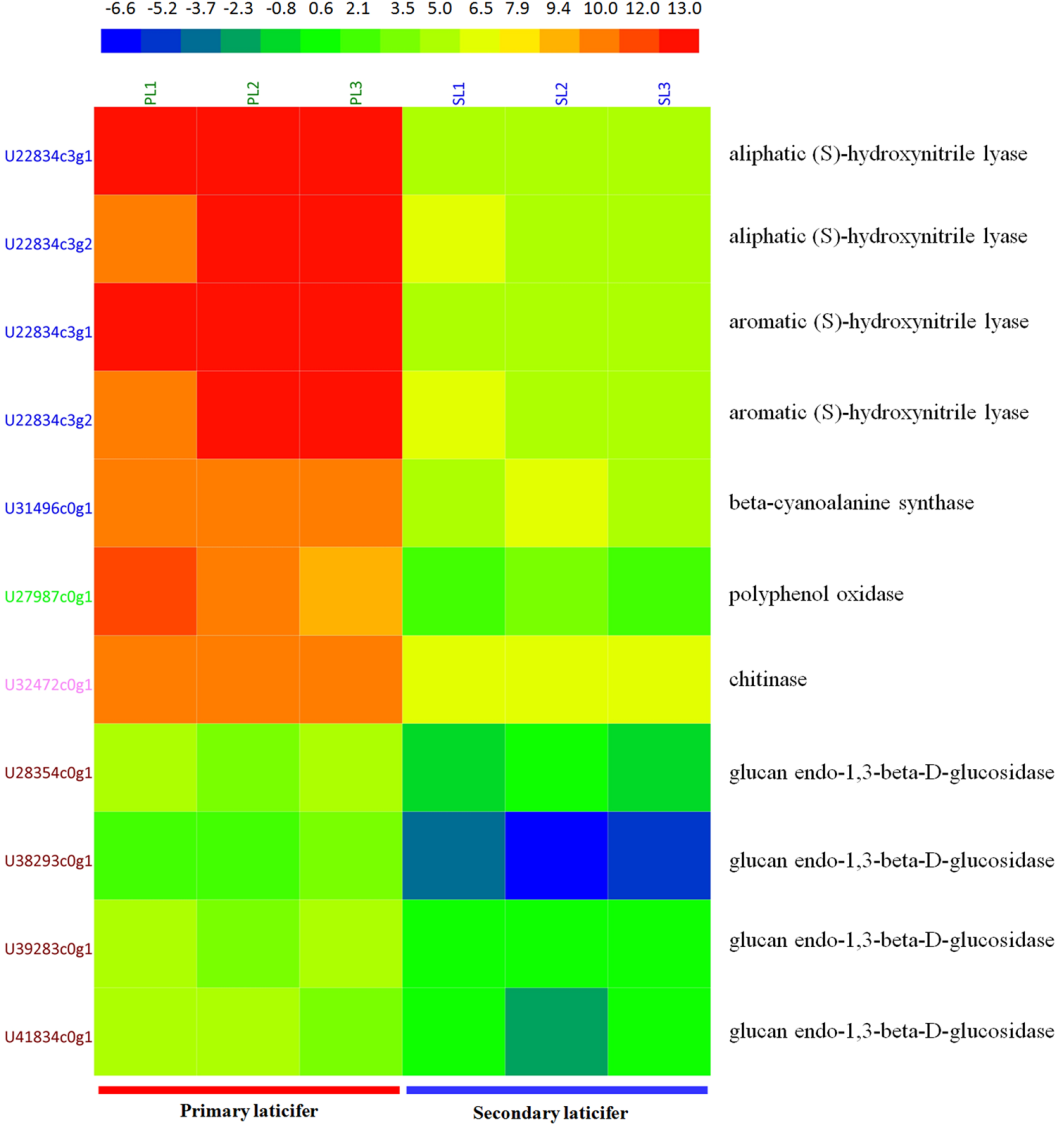
Heatmap of DEGs involved in biotic stresses. The expression levels of DEGs in TPM were compiled with Excel 2007, normalized using a log2 base method, and exported to the HemI toolkit^124^. The Heatmap was generated using the default parameters. The expression levels are presented in different colors, and the values are defined in the scale bar. PL, the primary laticifers; SL, the secondary laticifers.

The second most significant finding was the strong up-regulation of genes involved in defense against biotic stresses in the primary laticifers. These up-regulated genes included (S)-hydroxynitrile lyase, beta-cyanoalanine synthase, polyphenol oxidase, chitinase, and glucanase (Fig. 5), suggesting that the young tissue was prepared more adequately to defend attacks by herbivorous animals and pathogens.

The most significant up-regulated DEGs in the primary laticifers encoded (S)-hydroxynitrile lyases (EC4.1.2.47, HNL), including two aliphatic (S)-hydroxynitrile lyases (U22834c3g1, U22834c3g2) and two aromatic (S)-hydroxynitrile lyases (U22834c3g1, U22834c3g2). The TPM values of these genes were more than 17,000 in the primary laticifers, a value that is 200 to 400-fold higher than that in the secondary laticifers. HNL catalyzes the hydrolysis of (S)-hydroxynitriles and releases the toxin cyanide. The latter serves as a defense mechanism against herbivores and microbial attack in plants^29^. This report is the first to describe high expression of hydroxynitrilases in the primary laticifers. A hydroxynitrilase gene was originally cloned from the leaves of the rubber tree^30^. However, another DEG (U31496c0g1) encoded a beta-cyanoalanine synthase, which contributes to cyanide detoxification^31^. This DEG was up-regulated in the primary laticifers and had a TPM value of 2055.5, which is approximately 28-fold higher than that in the secondary laticifers (TPM = 72.9).

In addition, a polyphenol oxidase (PPO, U27987c0g1) was also highly up-regulated in the primary laticifers with an average TPM value of 2180.4 compared to a value of 7.1 in the secondary laticifers (Fig. 5). PPO has been demonstrated to function as a defense mechanism against pest insects and pathogens^32^.

Chitinase and glucan endo-1,3-beta-D-glucosidase (EC3.2.1.39) are hydrolases that are well known for their antifungal activity. A chitinase gene (U32472c0g1) was transcribed at very high levels in the primary laticifers and accounted for more than 95% of the total chitinase transcription in the primary laticifers with a TPM value of 3004.2, which was approximately 21-fold higher than that in the secondary laticifers (TPM = 139.4; Fig. 5). This chitinase has 311 amino acid residues and has only two C-terminal amino acid differences compared with hevamine (AJ007701) in rubber tree^33^. Five additional chitinase unigenes were also up-regulated in the primary laticifers; however, their expression levels were low (TPM < 100) compared to that of U32472c0g1. Thus, these genes were omitted from further analysis. None of the chitinase genes, however, were up-regulated in the secondary laticifers. Glucan endo-1,3-beta-D-glucosidase, also known as endo-1,3-beta-glucanase, laminarinase, and 3-beta-D-glucan glucanohydrolase, function in antifungal defense via degradation of fungal cell walls^34, 35^. More than 100 unigenes that were homologous to glucanase family members were identified in the laticifer transcriptomes, and of these, four were consistently up-regulated in the primary laticifers (FDR ≤0.001; log2Ratio ≥2; Fig. 5). None of the glucanase genes were consistently up-regulated in the secondary laticifers.

### Genes involved in the rubber biosynthesis pathway are mainly up-regulated in the primary laticifers

**Figure 6:**
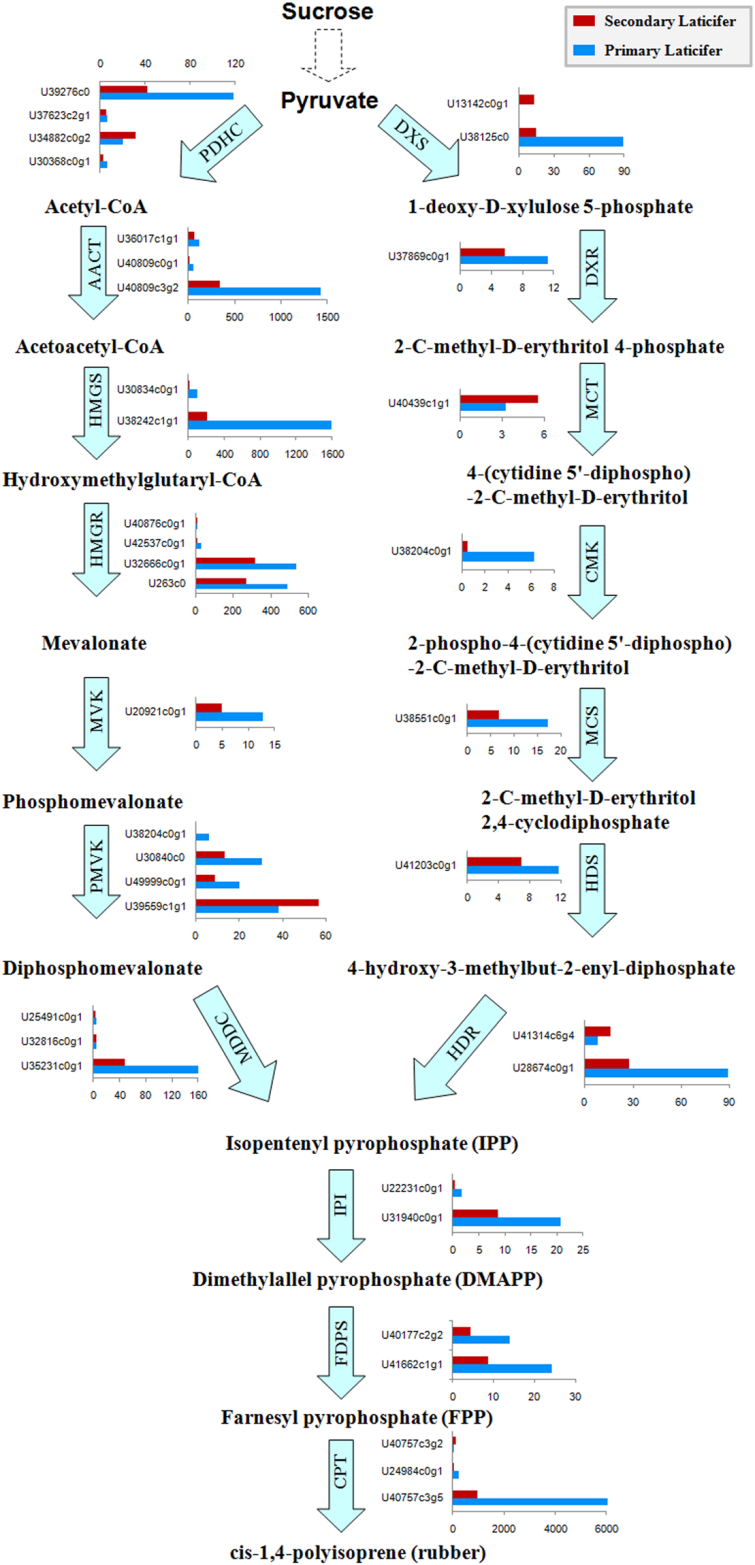
The rubber biosynthesis pathways and genes involved. The expression levels of each unigene in TPM are presented beside the enzymes.

Natural rubber is composed of mainly high molecular weight cis-polyisoprene synthesized via sequential condensation of isopentenyl diphosphate (IPP)^36^. IPP is synthesized via two pathways: the mevalonate (MVA) pathway, which occurs in the cytoplasm^37^, and the 2-C-methyl-D-erythritol 4-phosphate (MEP) pathway, which occurs in the plastid^38^. Almost all the enzymes involved in IPP synthesis were up-regulated in primary laticifer cells, except for the phosphomevalonate kinase (PMVK) gene (U39559c1g1) in the MVA pathway and the 2-C-methyl-D-erythritol 4-phosphate cytidylyltransferase (MCT) gene (U40439c1g1) in the MEP pathway (Fig. 6). Most genes that encode enzymes in the MEP pathway were transcribed at relatively low levels with TPM values <20, except for 1-deoxy-D-xyluloase 5-phosphate synthase (DXS, U38125c0) and 4-hydroxy-3-methylbut-2-en-1-yl diphosphate reductase (HDR, U28674c0g1), which had TPM values of approximately 90 in the primary laticifers (Fig. 6). The overall transcription levels of the genes in the conventional MVA pathway were much higher than those in the MEP pathway (Fig. 6). The most actively transcribed genes encoded key enzymes in the MVA pathway. These genes included hydroxylmethyglutaryl coenzyme A synthase (HMGS, U38242c1g1), two hydroxylmethyglutaryl coenzyme A reductases (HMGR, U32666c0g1 and U263c0), and acetyl coenzyme A acetyltransferase (AACT, U40809c3g2), with TPM values of 1600, 537, 489, and 1426, respectively, in the primary laticifer cells. These results suggest that the MVA pathway accounts for the majority of IPP biosynthesis.

Enzymes involved in post-IPP processes were also up-regulated in the primary laticifers (Fig. 6). Two unigenes (U31940c0g1 and U22231c0g1) that encode isopentenyl isomerase (IPI) were identified in both the primary and the secondary laticifers, and were up-regulated in the primary laticifers. The farnesyl diphosphate synthase (FDPS) family catalyzes the synthesis of farnesyl pyrophosphate (FPP), which subsequently serves as the priming substrate for the initiation of the prenyl chain. Thus, FDPS family members are crucial enzymes in the biosynthesis of rubber. Two FDPS family members, U40177c2g2 and U41662c1g1, were found to be up-regulated in the primary laticifers (Fig. 6). Furthermore, the cis-prenyltransferase (CPT) family, which is also known as *Hevea* rubber synthase (HRT), is crucial for integration of IPP units into the prenyl chain^39, 40^. U40757c3g5, which is the major transcript encoding CPT, was found to be up-regulated in the primary laticifers (Fig. 6).

### Genes involved in abiotic stress responses are up-regulated in the secondary laticifers

**Figure 7:**
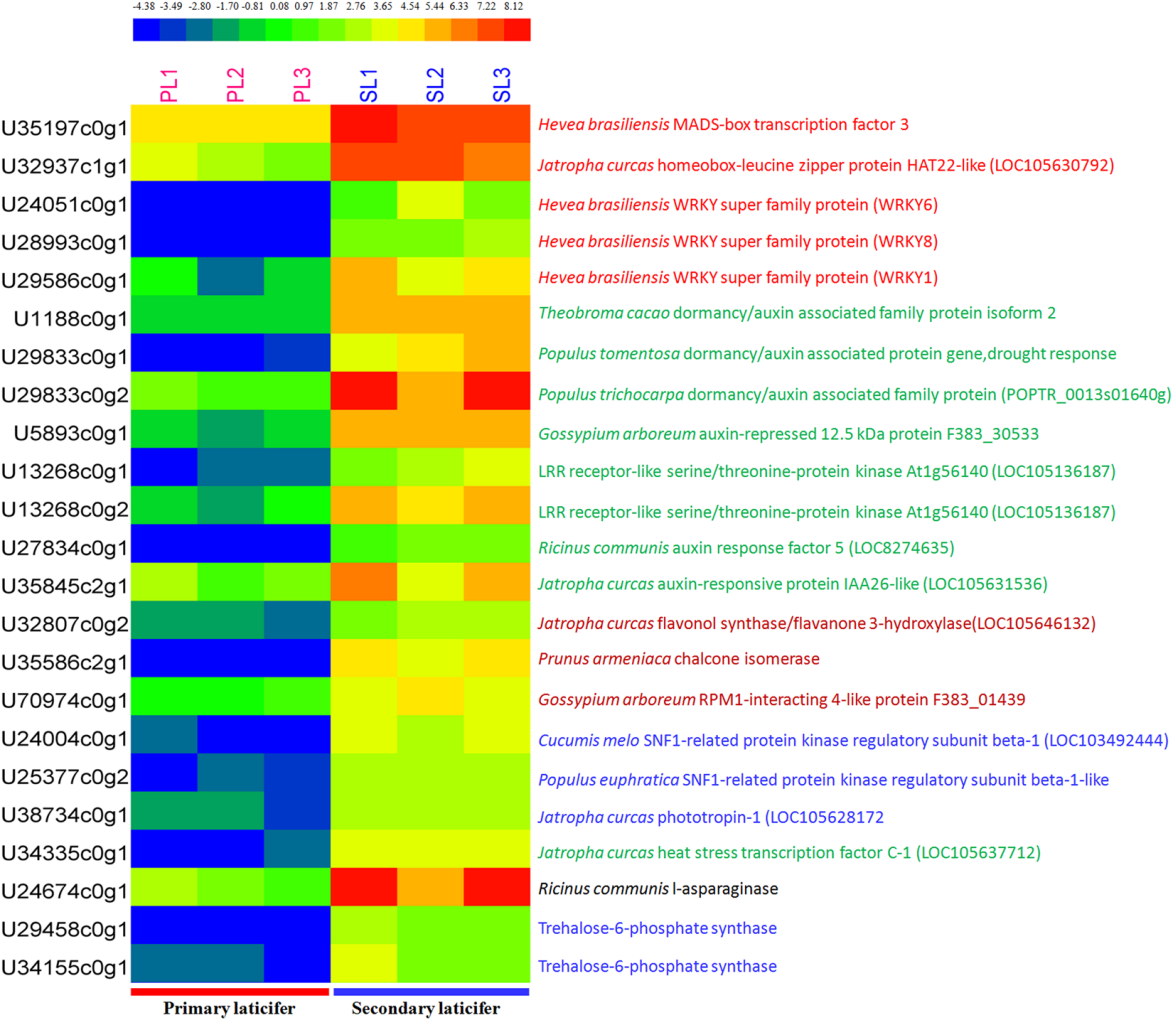
Heatmap of DEGs involved in abiotic stresses. The expression levels of DEGs in TPM were compiled with Excel 2007, normalized using a log2 base method, and exported to the HemI toolkit^124^. The figure was built using the default parameters. The expression levels are presented in different colors, and the values are defined in the scale bar. PL, the primary laticifers; SL, the secondary laticifers.

DEGs that were up-regulated in the secondary laticifers (FDR ≤0.001 and |Log2Ratio(RS/RP)| ≥2) were most related to abiotic stress responses, including transcription factors and/or signal receptors of brassinosteroid, karrikin, sucrose, and auxin. The most highly up-regulated transcription factors were a MADS-box transcription factor (U35197c0g1) and a homeobox-leucine zipper protein (U32937c1g1, Fig. 7). Other up-regulated factors included three WRKY superfamily proteins and eight auxin responsive proteins (Fig. 7). These DEGs may be related to the dormancy of the axillary buds in the tree trunk and may also be related to the dormancy of the secondary laticifers in untapped trees, resulting in the down-regulation of DEGs involved in rubber biosynthesis in the secondary laticifers (Fig. 6). Genes involved in rubber biosynthesis are indeed up-regulated by tapping^41^.

The trehalose-phosphate synthase (TPS) genes have been reported to enhance drought resistance^42, 43^. Two TPS (U29458c0g1 and U34155c0g1) genes were up-regulated in the secondary laticifers. A heat stress-responsive transcription factor C-1-like protein gene (U34335c0g1) was also up-regulated in the secondary laticifers. This protein homolog in Arabidopsis and rice is known to regulate the expression of heat shock proteins^44, 45^. Another DEG (U24674c0g1) encoding an asparaginase (EC 3.5.1.1) was also up-regulated in the secondary laticifers, and a homolog of this DEG (GmASP1) is known to be induced by cold stress in soybean^46^.

### Validation of representative DEGs by quantitative reverse-transcription polymerase chain reaction (qRT-PCR)

**Figure 8:**
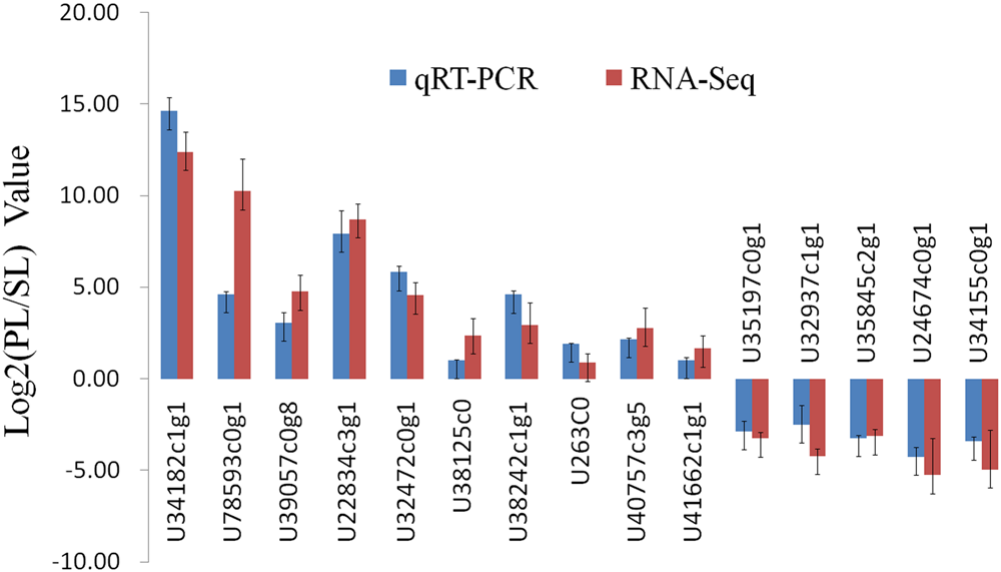
Quantitative RT-PCR validation of representative DEGs revealed by RNA-seq analysis.

To validate the RNA-seq results, 15 representative DEGs, including 10 up-regulated DEGs in the primary laticifers and five up-regulated DEGs in the secondary laticifers. Three reference genes that were stable in both laticifers (Supplementary Table S5) were selected for qRT-PCR analysis. The results indicated that the Log2(PL/SL) values of all the 15 DEGs (where PL is the primary laticifers and SL is the secondary laticifers) were similar between qRT-PCR and RNA-seq results (Fig. 8) with a correlation coefficient R^2^ of 0.876 (Supplementary Fig. S4), indicating that the RNA-seq comparative transcriptome analysis between the primary and the secondary laticifer cells was reliable.

## Discussion

### Comparison of laticifer paraffin sections versus laticifer isolation

We have compared the morphology of the primary and the secondary laticifers in both paraffin sections and via isolated laticifer networks. Paraffin sections are the traditional method widely used for studies of laticifer development in rubber tree^10^. When this method was used for morphological comparison between the primary and the secondary laticifers in this study, only the distribution patterns of the laticifer cells were found to be different. The primary laticifer cells were distributed at random among the parenchyma cells (Fig. 1b), whereas the secondary laticifer cells were distributed in rows or rings (Fig. 1d).

Isolated laticifers proved to be an ideal method to study the morphology and development of laticifers. This method was previously reported in 1987^47^. We modified this method by inclusion of a fixation step using FAA solution before boiling and by staining the isolated laticifer networks using iodine bromide solution instead of Sudan solution, which greatly improved the quality of the images. Based on our observation, the secondary laticifer cells had straight and smooth cell walls (Fig. 2h,i,j). The neighboring tubes were anastomosed by bridges, and each laticifer cell was joined to two or more cells of the neighboring tubes at different locations (Fig. 2h,i,j). These observations did not require expensive equipment, and the quality of these results were as good as previous observations gained with spectral confocal laser scanning microscopy^48^. Moreover, the detailed laticifer structure of the primary laticifer network had never been revealed. We isolated the primary laticifer elements ranging from the meristem of young shoots to the mature region of branches and observed the sequential development and intrusive growth of the primary laticifers (Fig. 2a,b), as this growth was quite different from that of the secondary laticifer cells. The most impressive morphology of the primary laticifer vessels is the necklace-like structures (Fig. 2e) that are formed during intrusive growth. The neighboring “beads” then merged to form laticifer tubes of larger diameter. The neighboring primary laticifer tubes were connected by bridges to form a laticifer network (Fig. 2f,g), which is similar to the secondary laticifer network.

### Necklace-like morphology of the primary laticifers is facilitated by over-expression of cell wall process genes

The rubber latex collected from rubber tree, either from the primary laticifers or from the secondary laticifers, is the cytoplasm of laticifer cells^8^ and contains the transcriptome of the laticifer cells. Therefore, the rubber latex provides a convenient material to study the transcriptome profiles of the single cell types. In order to collect the latex, however, the bark of the rubber trees was cut, and the neighboring cells of the laticifer cells were also broken, contributing to possible contamination of the latex. To reduce this potential contamination, the latex flow of the first 5 min for the secondary laticifers or the first droplet for the primary laticifers was discarded. To further eliminate contaminated RNA, a threshold cut-off TPM >1 was arbitrarily used to minimize false-positive gene expression data.

Through comparative analysis, we found that the primary and secondary laticifers exhibited distinct expression profiles. One of the most significant differences was that DEGs involved in cell wall processes were up-regulated in the primary laticifers (Fig. 4), corresponding to the necklace-like morphology of these cells. We identified eight pectinesterase genes that were highly up-regulated in the primary laticifers (Fig. 4). Pectinesterase is a ubiquitous cell wall-associated enzyme that facilitates pectin de-esterification and promotes pectin degradation especially during fruit ripening^19, 20^. Mutational disruption of the pectin methylesterase 3 (AtPME3) gene in *Arabidopsis thaliana* resulted in changes in pectin methylesterification status and caused hypersensitivity towards the specific interference of Zn^+2^ with cell wall-controlled growth processes^49^. Over-expression of a fungal pectin methylesterase gene in tobacco led to alterations in cell wall metabolism and a dwarf phenotype^50^. The strong up-regulation of pectinesterase in the primary laticifers may play a major role in the intrusive growth and the formation of necklace-like structures of the primary laticifers (Fig. 2).

In addition to pectinesterase genes, DEGs related to plant-type cell wall construction were found to be highly up-regulated in the primary laticifers (Fig. 4). These genes include berberine bridge enzyme, class III peroxidase, alpha-expansin, RALF-like protein, subtilisin-like protease, alpha-glucosidase, and heparanase-like protein. Berberine bridge enzyme (BBE)-like enzymes has recently been demonstrated to play a role in plant cell wall metabolism via oxidation of monolignol^51^. *Arabidopsis* Class III peroxidase (AtPRX71) was shown to strengthen cell walls and restrict cell expansion during normal growth and in response to cell wall damage^52^. Alpha-expansin is a well-known cell wall component that plays a key role in “acid-growth” of plant cells^53^. Rapid alkalinization factor (RALF) is a secreted peptide that mediates a rapid alkalinization of extracellular space by mediating a transient increase in the cytoplasmic Ca^2+^ concentration, leading to calcium-dependent signaling events and regulation of cell expansion^54, 55^. Disruption of the kexB gene that encodes a subtilisin-like processing protease in *Aspergillus oryzae* caused a decrease in cell wall alpha-1,3-glucan and substantial morphological defects^56^. Furthermore, a subtilisin-like serine protease was found to be essential for mucilage release from *Arabidopsis* seed coats^57^. Heparanase-like protein, an endoglycosidase, is a cell surface protein and an extracellular matrix-degrading enzyme that catalyzes the hydrolysis of heparan sulfate proteoglycans (HSPGs) into heparan sulfate side chains and core proteoglycans, and may have a role in cell growth regulation^58–60^.

DEGs that were up-regulated in the primary laticifers also included genes related to lignin metabolic processes in the cell wall, and such genes included a laccase, a transaldolase, and three xylem serine protease genes. Laccase activity is important to cell wall reconstitution in regenerating protoplasts^61^, and downregulation of laccase caused alterations in phenolic metabolism and cell wall structure in *Populus trichocarpa* ^62^. Additionally, knockdown of a laccase in *Populus deltoides* altered cell wall chemistry and increased sugar release^63^. Transaldolase is an enzyme of the pentose phosphate pathway and plays an important role in lignin biosynthesis^64, 65^ and biocontrol function^66^. Differential expression of xylem serine protease genes has been shown to be one of the major causes of ruptured cell walls in the defective seed coats of a soybean mutant^67^.

DEGs that were clustered in the cell size-regulation category encoded two metallothionein 3-like proteins, a WAT1-like protein, and a glucan endo-1, 3-beta- glucosidase. Metallothionein -like proteins, which are metal-binding proteins, function as detoxifier and are up-regulated in animal and plant cells upon exposure to heavy metals^68–71^. However, they are also involved in fruit ripening of strawberries^72^, post-harvest senescence of *Alstroemeria* flowers^73^, and somatic embryogenesis of white spruce^74^, albeit via an unknown mechanism. In *Arabidopsis thaliana* WAT1 encodes a plant-specific, predicted integral membrane protein, and its expression is preferentially associated with vascular tissues, including developing xylem vessels and fibers^75^. Mutation of WAT1 in *Arabidopsis* results in cell elongation defect^75^. Glucan endo-1, 3-beta- glucosidase is an important component of plant cell walls^76^ and plays a role in defense against fungal pathogens^77^. It also plays a role in compression stress resistance, because lowering the amount of callose results in reduced cellular stiffness and increased viscoelasticity of pollen tubes^78^. The up-regulation of glucan endo-1,3-beta-glucosidase in the primary laticifers suggest some similarities in the growth patterns of primary laticifers and pollen tubes.

Interestingly, three DEGs that were highly up-regulated in the primary laticifer cells were clustered in the categories of pollen tube guidance and regulation of pollen growth, and these genes were an annexin-like protein (U39057c0g8), a nucleoredoxin-like protein (U31194c0g1), and an early nodule-specific protein homolog (*Hev b 13*, U78593c0g1). Annexins interact with cell-membrane components that are relevant to the structural organization of the cell and to changes in the cell shape, intracellular signaling, growth control, and these proteins also act as atypical calcium channels^23, 24^. Annexins have been implicated in regulation of pollen tube and fiber growth in *Arabidopsis* and cotton^21, 22^. Nucleoredoxin functions as a redox-dependent negative regulator and/or a transcriptional regulator^79–81^, and is critical for growth of pollen tubes in pistil of *Arabidopsis* ^82^. The early nodule-specific protein homolog (*Hev b 13*) is an allergenic lipolytic esterase and is homologous to patatin^83^, a potato (*Solanum tuberosum*) protein. Patatin is thought to be a storage protein that mainly localizes to the vacuole of the potato tube^84, 85^; however, it also has lipid acyl hydrolase activity^85^. The patatin-like protein in rubber laticifers was not localized to the vacuole^86^, and exhibited lipid acyl-transferase and PLA2-like activity^87^. As a result, this protein may catalyze the cleavage of fatty acids from membrane lipids and thus may function in the reconstruction of the laticifer membrane and/or defense against pests. Moreover, a patatin-like protein was found to be critical to the programmed degeneration of the *Arabidopsis* sporophytic tapetum to support pollen development^88^. These data further suggest that the development and growth of the primary laticifer cells may have some similarities to pollen grains and pollen tubes.

Two DEGs that were clustered in the category of cell wall macromolecule catabolic processes included a lysin motif (LysM) domain receptor-like kinase 3 (Lyk3, U28972c0g1) and a wall-associated receptor kinase (U38208c0g2). Lyk3 may recognize microbe-derived N-acetylglucosamine (NAG)-containing ligands and regulate the cross talk between immunity and abscisic acid responses in *Arabidopsis* ^89^. Furthermore, this protein was found to be an entry receptor in rhizobial nodulation factor signaling in Medicago^90^, and its localization and dynamics were altered in response to symbiotic bacteria^91^. Wall-associated kinase (WAK) and WAK-like kinase (WAKL) constitute a family of receptor-like kinase genes that encode transmembrane proteins^92^, and some of the members were co-expressed with pathogen defense related genes^93, 94^, whereas others were required for cell elongation and plant development^92^.

Taken together, these data demonstrate a considerable number of DEGs that are related to cell wall modification and cell development were up-regulated in the primary laticifers, resulting in the active intrusive growth of the primary laticifer cells and the formation of necklace-like morphology.

### Up-regulation of lytic enzymes in the primary laticifers suggests a role for defense against biotic stresses

We have demonstrated that many genes that function in defense against biotic stress, especially genes that encode lytic enzymes, are highly up-regulated in the primary laticifers. These genes include (S)-hydroxynitrile lyase, beta-cyanoalanine synthase, polyphenol oxidase, chitinase, and glucanase (Fig. 5). Among these genes, (S)-hydroxynitrile lyase genes (HNL) were the most significant DEGs. HNL catalyzes the hydrolysis of (S)-hydroxynitriles and releases toxic cyanide, which serves as a defense mechanism against herbivores and microbial attack in plants^29^. The (S)-hydroxynitrile lyase gene was first cloned by Hasslacher and colleagues from the leaves of *Hevea brasiliensis* ^30^ and was over-expressed heterologously in *Escherichia coli* and *Saccharomyces cerevisiae* ^95^. Our report is the first to demonstrate that the (S)-hydroxynitrile lyase gene was highly up-regulated in the primary laticifer cells than in the secondary laticifer cells. Protease inhibitors are known as defense proteins and have been over-expressed in crops for herbivore pest control^96^. Chitinases have been identified in the laticifers of numerous latex-containing species, including monocotyledons, dicotyledons, and a gymnosperm^97–100^. These enzymes were also identified at high levels in the laticifer cells of rubber tree^101^. Microbial chitinases have been shown to exhibit antifungal activity and used for biological control against plant fungal diseases^102–105^. Chitinases of insect origin also exhibit toxic effects on other insects upon oral ingestion^106, 107^. In latex-containing plants, jasmonic acid, a plant hormone that is involved in signal transduction of plant responses to herbivory, was proven to induce expression of chitinases in the latex of *Ficus carica* ^108^, and expression of chitinases in the latex of *C*. *papaya* was found to be induced by wounding^109^. Furthermore, a poplar chitinase was over-expressed in tomato and inhibited the development of the Colorado potato beetle^110^. These results suggest that chitinases in latex may play a defensive role. Because chitin is the major component of the cell walls of fungi and some insects, it is reasonable to speculate that chitinases in the laticifer cells of rubber trees may protect the plants from fungal infection and insect attacks.

The laticifer cells of the rubber tree are dedicated primarily to the biosynthesis of rubber (*cis-*1,4-polyisoprene); thus, the most highly expressed genes in the laticifer cells are likely to be genes involved in the biosynthesis of rubber. Indeed, gene transcripts encoding enzymes involved in rubber biosynthesis are 20− to 100−fold higher in laticifers than in leaves^111^; however, several putative defense genes, including chitinases, pathogenesis-related proteins, phenylalanine ammonia-lyase, chalcone synthase, chalcone isomerase, and 5-enolpyruvylshikimate 3-phosphate synthase, are also expressed at 10− to 50−fold higher levels in laticifers than in leaves^111^. Chitinases/lysozymes are the most abundant proteins in the latex, are localized primarily in the lutoid, and account for approximately 20% of the total soluble protein of the latex^101^. The above findings are all based on analysis of the secondary laticifers. Here, we compared the transcriptional profiles of the primary and the secondary laticifer cells and revealed that the expression levels of (S)-hydroxynitrile lyases, chitinases, glucanases, and protease inhibitors are up-regulated in the primary laticifer cells. Confirmation of these findings via qRT-PCR supports this comparison and suggests that the primary laticifer cells and/or the young tissues are more adequately prepared to defend against attacks by herbivorous animals and pathogens than the secondary laticifers.

### Up-regulation of DEGs in the secondary laticifers suggests defense against abiotic stresses

We have demonstrated that the secondary laticifer cells of untapped rubber trees display features of dormancy and abiotic stress resistance. DEGs that were up-regulated in the secondary laticifers include transcription factors and signal receptors of brassinosteroid, karrikin, sucrose, and auxin (Fig. 7), which are related to dormancy. This differential expression may have resulted in the down-regulation of DEGs involved in rubber biosynthesis (Fig. 6). The obviously abiotic stress-related DEGs that were up-regulated in the secondary laticifers include trehalose-phosphate synthase genes, heat stress-responsive transcription factor C-1 genes, and an asparaginase gene (Fig. 7). Trehalose-phosphate synthase genes have been reported to enhance drought resistance^42, 43^, while the heat stress-responsive transcription factor C-1 in *Arabidopsis* and rice regulates the expression of heat shock proteins^44, 45^. In addition, an asparaginase (GmASP1) in soybean is induced by cold stress^46^. Therefore, the rubber tree asparaginase homolog is potentially related to cold stress. Taken together, these findings indicate that the secondary laticifer cells are more adequately prepared to defend against abiotic stresses than the primary laticifers.

### Rubber biosynthesis is more active in the primary laticifers of the untapped rubber trees

The latex tapped from the secondary laticifer cells of the trunk of the rubber trees (>6 years old) were the major source of natural rubber, however, the primary laticifer cells were actually more active in rubber biosynthesis in the untapped rubber trees (Fig. 6). This observation does not mean that the secondary laticifer cells are not as productive as the primary laticifer cells. The down-regulation of the rubber biosynthesis genes in the secondary laticifer cells (Fig. 6), however, may be explained by the dormancy of the secondary laticifer cells induced by dormancy-related genes (Fig. 7), since the secondary laticifer tubes may be full of rubber particles in trees that have not been tapped for 6 years. Previous evidence has suggested that tapping stimulates rubber biosynthesis in the secondary laticifer cells^41, 112^.

In conclusion, the primary and the secondary laticifer cells are morphologically and functionally distinct. Genes involved in cell wall modification were highly up-regulated in the primary laticifers, and correspond to the necklace-like morphology of the primary laticifer tubes. By contrast, the cell walls of the secondary laticifer cells were relatively smooth and straight. The primary and the secondary laticifer cells are also functionally different. Genes involved in defense against biotic stresses were highly up-regulated in the primary laticifer cells, whereas genes involved in abiotic stresses and dormancy were highly up-regulated in the secondary laticifer cells. These findings suggest that the primary laticifer cells are more adequately prepared to defend against biotic stresses such as insects and pathogens, while the secondary laticifer cells are more adequately prepared to defend against abiotic stresses. Interestingly, genes involved in rubber biosynthesis were highly up-regulated in the primary laticifers compared with those in the secondary laticifer cells. These observations could be explained by feedback inhibition in the secondary laticifer tubes, because the secondary laticifer cells in trees that have not been tapped for 7 years may be full of rubber particles. Taken together, these findings indicate that the two types of laticifers are morphologically and functionally distinct.

## Methods

### Histochemical analysis of the primary and the secondary laticifers

Rubber trees (*Hevea brasiliensis* Mull. Arg.) used in this study were grafted plants of clone RY7-33-97 using the seedlings of RY7-33-97 as rootstock. The plants were grown on the experimental farm of the Chinese Academy of Tropical Agricultural Sciences in Danzhou City, Hainan Province for 7 years without tapping. Histochemical observation of cross-sections of the bark of the tree trunks and young branches was performed as previously described^113^ with a few modifications. The bark samples of the tree trunks and epicormic young branches as shown in Fig. 1 were excised and fixed in FAA solution (5 mL formalin, 5 mL glacial acetic acid, 90 mL 70% ethanol) for 3 d. After dehydration in an ethanol/n-butyl alcohol series, the samples were treated with an iodine and bromine solution (5 g iodine, 0.4 mL bromine, and 100 mL glacial acetic acid)^10^ for 36 h at 60 °C. The samples were washed with glacial acetic acid, dehydrated in an *n*-butyl alcohol series, and embedded in paraffin. Sections of 10 µm were prepared with a rotary microtome (LEICA RM2245, Wetzlar, Germany), dewaxed in xylene, and stained with fast green solution (5 g of fast green in 100 ml of 95% ethanol). The sections were viewed and photographed with a light microscope (Axioskop 40, Zeiss, Oberkochen, Germany).

To study the three-dimensional morphology of the laticifer networks, the primary and the secondary laticifer networks were isolated using a previously described method^47^ with a few modifications. The bark samples of the tree trunks and segments of the young branches from three 7-year-old untapped rubber trees as shown in Fig. 1 were fixed in FAA solution for 24 h, boiled in 10% KOH for 20 min as previously described^47^, and then rinsed in running water for 2 h. The laticifer networks were dissected from the samples under a dissecting microscope by removing the peridermal layers and the parenchyma cells surrounding the laticifer cells with a pair of forceps and a dissecting needle. The laticifer cells were then stained with iodine bromide solution^114^ and observed and photographed with a light microscope.

### Latex collection and total RNA extraction

A total of 18 healthy untapped 7-year-old rubber trees (clone RY7-33-97) were randomly selected, in which nine were used for collecting primary latex and the other nine were used for collecting secondary latex. Three independent latex replicates were collected from each of the primary and the secondary laticifer cells during different seasons (July 2014, October 2014, and April 2015) to minimize on the possible influence of seasonal changes in the transcriptome profiles, and each sample contained the latex of the primary or the secondary laticifer cells of three trees. The latex of the secondary laticifer cells was collected by tapping the bark of the trunk 1 m above the ground (Fig. 1a). To reduce contamination by non-laticifer RNAs from neighboring cells, the latex flow of the first 5 min was discarded, and the remaining latex was collected in 50-mL centrifuge tubes on ice. To collect latex from the primary laticifer cells, the barks of the epicormic shoots (Fig. 2b) were cut with a sharp scalpel. To reduce contamination by non-laticifer RNAs from neighboring cells, the first drop of latex was discarded, and the remaining latex was collected in 1.5-mL centrifuge tubes and stored on ice. Total RNA was extracted as previously described^115^. The quality of the total RNA was evaluated using a 2100 Bioanalyzer (Agilent Technologies, Santa Clara, CA, USA).

### Construction of cDNA library and sequencing

Poly(A) mRNA was enriched from total RNA using magnetic beads with Oligo (dT) (Dynabeads, Invitrogen, Carlsbad, CA, USA). The enriched mRNA was randomly broken into short fragments and reverse transcribed to cDNA using the Superscript III First-Strand Synthesis System (Invitrogen). Double-strand (ds) cDNA was synthesized using DNA polymerase I and RNase H (TaKaRa, Dalian, China), and purified with an Agencourt AMpure XP Kit (Beckman Coulter, Beijing, China). Adapters were then added to the two ends of the ds cDNA, and molecules were then amplified by PCR to construct the sample library. The quality of the libraries was evaluated by Agilent 2100 Bioanaylzer and ABI StepOnePlus Real-Time PCR System. The libraries of replicate I were sequenced using Illumina HiSeq™ 2000 with an average read length of 90 nt (BGI, Shenzhen, China). Replicates II and III were sequenced using HiSeq2500 with an average read length of 100 nt (Biomarker, Beijing, China).

### De novo sequence assembly, annotation, and classification

Raw reads that had more than 10% of bases with low-quality scores (Q < 20) and those that still contained adaptors and unknown nucleotides of more than 5% were filtered with Filter_fq software. *De novo* assembly of the clean reads was performed using Trinity Version 2.1.1^16^, according to the recommended protocol. In addition, bowtie V1.1.2^17^, a plug-in program in the Trinity software, was used.

The unigenes were annotated by homology searches against the public databases, including the non-redundant NCBI peptide database (NR, http://www.ncbi.nlm.nih.gov), Swiss-Prot (http://www.expasy.ch/sprot/), the Kyoto Encyclopedia of Genes and Genomes (KEGG, http://www.genome.jp/kegg/), and the Cluster of Orthologous Groups (COG, http://www.ncbi.nlm.nih.gov) using the BLASTX software^116^ with a cut-off value of 1E-5. The unigenes were also screened against the NCBI nucleotide database (NT) using the BLASTN program. The best alignment results were accepted for annotation of the unigenes.

To perform functional classification of the unigenes, GO annotation was carried out using the Blast2GO program (http://www.geneontology.org) based on the molecular function, biological process, and cellular component.

### Mapping and detection of DEGs

The eXpress method^117^ was used to estimate gene abundance. The expression values of reads were normalized using the trimmed mean of M values (TMM) method^118, 119^. To minimize the detection of false positives in gene expression of the laticifer cells, a threshold cut-off of TPM >1 was arbitrarily used to identify genes that were expressed in at least one sample. To identify DEGs, the raw counts were examined with EdgeR using standard parameters^18^, and an adjusted *p*-value cut-off ≤0.001 was used for FDR.

### Functional enrichment analysis for DEGs

Before GO enrichment analysis, a GO annotation list of each unigene was generated using the Blast2GO program, and genes were grouped into categories based on biological properties according to the data of “go_201603-termdb-data.gz” in the GO database. A GO-Seq package^120^ was utilized to analyze the enriched and depleted GO categories for genes that were either up-regulated or down-regulated in the pair-wise DE analysis, a *p*-value cut-off of 0.05 was used.

Additionally, to gain insight into the pathways altered between the primary and the secondary laticifer cells, DEGs were annotated and imported to MapMan^121^ for visualization.

### Validation of representative DEGs by qRT-PCR

Real-time PCR was used to validate transcriptome analysis results in accordance to MIQE guideline^122^. We selected fifteen genes with significant differential expression levels and important biological functions for real-time PCR confirmation. The stable reference genes of a ubiquitin-protein ligase (HQ323249), eIF1Aa (HQ268022), and YLS8 (HQ323250)^123^, were used to normalize the expression levels. The primers (Supplementary Table S5) were designed by Primer Premier 5.0 software and were verified by sequencing their amplified fragments. Rubber latex of the primary and the secondary laticifers were collected as described above with three biological replicates. Total RNA was extracted as previously described^115^. The quality of the total RNA was evaluated using a 2100 Bioanalyzer (Agilent Technologies, Santa Clara, CA, USA). The RNA integrity number (RIN) values of the RNA samples are provided in Supplementary Table S6. First-strand cDNA was synthesized using the Revertaid™ First Strand cDNA Synthesis Kit (Fermentas, Canada). The efficiency of each primer pair was estimated as ranging from 1.89 and 1.99. Then, qRT-PCR was performed with the SYBR^®^ Premix Ex Taq^TM^ II Kit (Takara, Japan) according to the manufacturer’s protocol. A 20-μl reaction mix composed of 10 μl SYBR^®^ Premix Ex Taq II (2x), 0.4 μl ROX Reference Dye (50x), 100 ng of template cDNA, 0.8 μl PCR forward primer (10 μM), and 0.8 μl PCR reverse primer (10 μM) was used. The reaction was performed in a Rotor Gene6000 (Corbett Research, Sydney, Australia) using the following parameters: predenaturation at 95 °C for 30 s, followed by 40 cycles of denaturation at 95 °C for 5 s, annealing at 58 °C for 30 s, and elongation at 72 °C for 30 s. The experiments were performed with three biological replicates and three technique replicates. The relative expression levels of the target genes were determined using the 2^−ΔΔCt^ method^125^. The significance of the correlation between the RNA-Seq and qRT-PCR results was analyzed using IBM SPSS Statistics Version 24, and the correlation curves were created with Excel 2007 (Microsoft Inc., USA).

## Electronic supplementary material

Supplementary Figures

Supplementary Table S1

Supplementary Table S3

Supplementary Table S4

Supplementary Table S5

Supplementary Table S6

Supplementary Table S7
