# Supplementary material for: Comparative morphology and transcriptome analysis reveals distinct functions of the primary and secondary laticifer cells in the rubber tree
Source: Sci Rep. 2017 Jun 9;7:3126. doi: 10.1038/s41598-017-03083-3 (PMC5466658; doi:10.1038/s41598-017-03083-3)
Supplement: Supplementary file 1 — Supplementary Figures [file 41598_2017_3083_MOESM1_ESM.pdf]

Supplementary Information 1

Comparative morphology and transcriptome analysis reveals distinct functions of the primary and secondary laticifer cells in the rubber tree

Deguan Tan[1, *], Xiaowen Hu[2,*], Lili Fu[1,*], Anuwat Kumpeangkeaw[1,3], Zehong Ding[1], Xuepiao Sun[1], Jiaming Zhang[1]

[1]Institute of Tropical Bioscience and Biotechnology, MOA Key Laboratory of Tropical Crops Biology and Genetic Resources, Hainan Bioenergy Center, CATAS, Xueyuan Road 4, Haikou, Hainan Province, 571101, China. [2]Zhanjiang Experimental Station, CATAS, West Libration Road 20, Zhanjiang, Guangdong Province, 524013, China. [3]Song Khla Rubber Research Centre, Department of Agriculture, Ministry of Agriculture and Cooperatives, Had Yai, Song Khla, 90110, Thailand.

*These authors contributed equally to this work.

Correspondence and requests for materials should be addressed to J.Z. (zhangjiaming@itbb.org.cn)

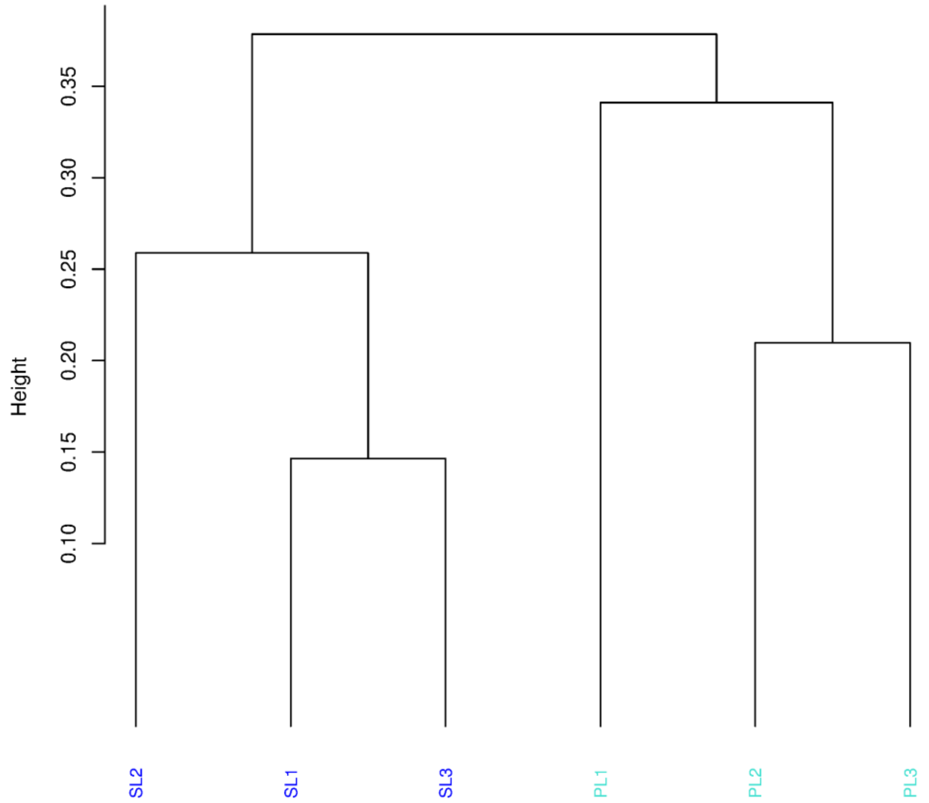

Supplementary Figure S1. Sample-clustering of the transcriptome replicates of the primary laticifer cells (PL) and the secondary laticifer cells (SL). The tree was constructed based on the Pearson correlations of gene expression values in each sample.

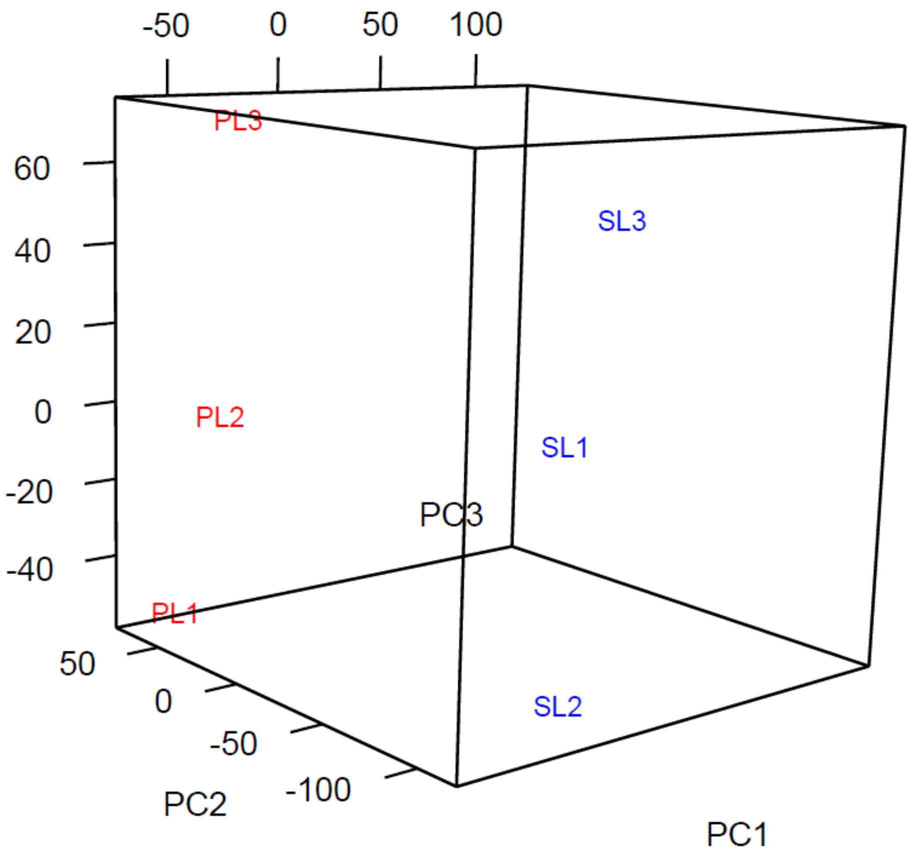

Supplementary Figure S2. PCA plot analysis of the transcriptome data. The transcriptome profile replicates of the primary laticifer cells (PL) and the secondary laticifer cells (SL) are well separated. The analysis was performed with 'prcomp' function in R software.

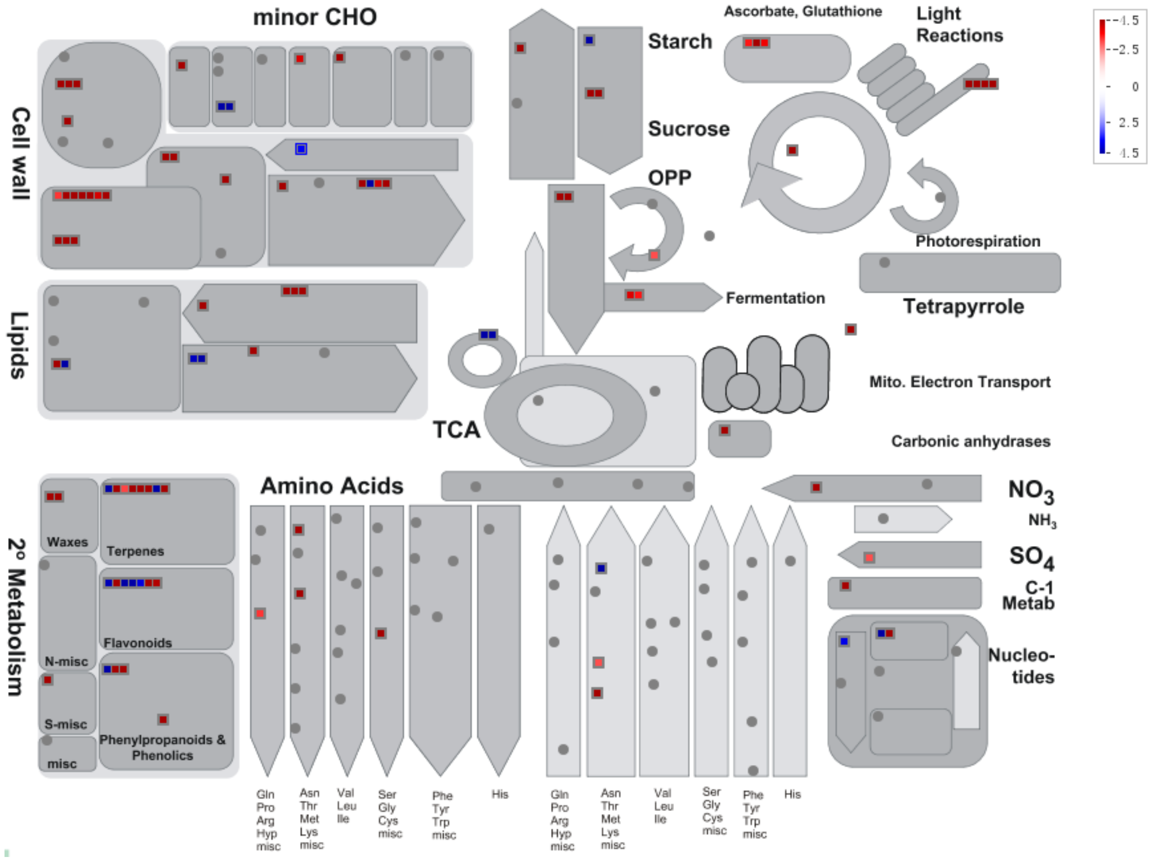

Supplementary Figure S3. Metabolism overview of genes differentially expressed between the primary and secondary laticifer cells. The analysis was performed in the MapMan software, and log2(SL/PL) values were used. Blue indicates up-regulation in gene expression, and red indicates down-regulation.

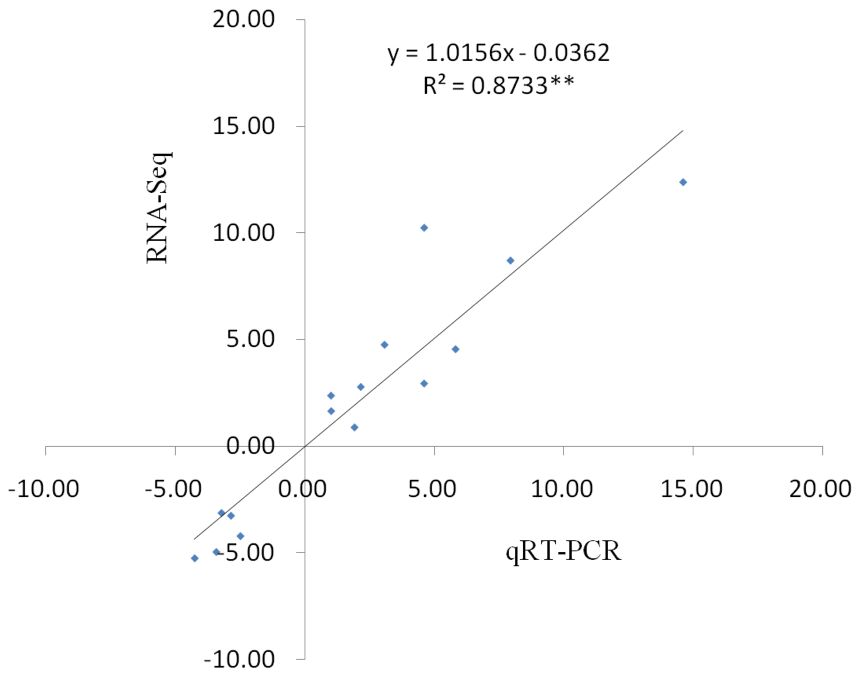

Supplementary Figure S4. Correlation analysis of the expression levels of representative genes as estimated by RNA-Seq and qRT-PCR methods. The significance of correlation was analyzed using IBM SPSS Statistics Version 24, and the correlation curves were created with Excel 2007 (Microsoft Inc., USA).
