# Supplementary material for: Comparative morphology and transcriptome analysis reveals distinct functions of the primary and secondary laticifer cells in the rubber tree
Source: Sci Rep. 2017 Jun 9;7:3126. doi: 10.1038/s41598-017-03083-3 (PMC5466658; doi:10.1038/s41598-017-03083-3)
Supplement: Supplementary file 2 — Supplementary Table S1 [file 41598_2017_3083_MOESM2_ESM.doc]

**Supplementary Table S1. Statistic data of the sequencing project**

| Total raw reads | Total clean reads | clean nucleotides (nt) | Unigene number | GC (%) | Transcripts | | |
| --- | --- | --- | --- | --- | --- | --- | --- |
|  |  |  |  |  | number | Average length (nt) | N50 (nt) |
| 312,566,596 | 261,566,596 | 30,923,276,992 | 103,704 | 41.54 | 131,078 | 783 | 1,406 |
