# Supplementary material for: Comparative morphology and transcriptome analysis reveals distinct functions of the primary and secondary laticifer cells in the rubber tree
Source: Sci Rep. 2017 Jun 9;7:3126. doi: 10.1038/s41598-017-03083-3 (PMC5466658; doi:10.1038/s41598-017-03083-3)
Supplement: Supplementary file 6 — Supplementary Table S6 [file 41598_2017_3083_MOESM6_ESM.doc]

Supplementary Table S6. RIN values of RNA isolated from the primary and secondary latex

| Laticifer type | Replicate | RIN value |
| --- | --- | --- |
| Primary laticifer (PL) | PL1 | 7.4 |
|  | PL2 | 8.6 |
|  | PL3 | 8.5 |
| Secondary laticifer (SL) | SL1 | 8.5 |
|  | SL2 | 7.7 |
|  | SL3 | 7.6 |
